# Petri nets and ODEs as complementary methods for comprehensive analysis on an example of the ATM–p53–NF-$\kappa$B signaling pathways

**DOI:** 10.1038/s41598-022-04849-0

**Published:** 2022-01-21

**Authors:** Kaja Gutowska, Daria Kogut, Malgorzata Kardynska, Piotr Formanowicz, Jaroslaw Smieja, Krzysztof Puszynski

**Affiliations:** 1 Institute of Computing Science, Poznan University of Technology, Piotrowo 2, 60-965 Poznan, Poland; 2 Department of Biosensors and Processing of Biomedical Signals, Silesian University of Technology, Roosevelta 40, 41-800 Zabrze, Poland; 3 Department of Systems Biology and Engineering, Silesian University of Technology, Akademicka 16, 44-100 Gliwice, Poland; 4 Institute of Bioorganic Chemistry, Polish Academy of Sciences, Noskowskiego 12/14, 61-704 Poznan, Poland

**Keywords:** Cellular signalling networks, Computational models, Gene regulatory networks

## Abstract

Intracellular processes are cascades of biochemical reactions, triggered in response to various types of stimuli. Mathematical models describing their dynamics have become increasingly popular in recent years, as tools supporting experimental work in analysis of pathways and regulatory networks. Not only do they provide insights into general properties of these systems, but also help in specific tasks, such as search for drug molecular targets or treatment protocols. Different tools and methods are used to model complex biological systems. In this work, we focus on ordinary differential equations (ODEs) and Petri nets. We consider specific methods of analysis of such models, i.e., sensitivity analysis (SA) and significance analysis. So far, they have been applied separately, with different goals. In this paper, we show that they can complement each other, combining the sensitivity of ODE models and the significance analysis of Petri nets. The former is used to find parameters, whose change results in the greatest quantitative and qualitative changes in the model response, while the latter is a structural analysis and allows indicating the most important subprocesses in terms of information flow in Petri net. Ultimately, both methods facilitate finding the essential processes in a given signaling pathway or regulatory network and may be used to support medical therapy development. In the paper, the use of dual modeling is illustrated with an example of ATM/p53/NF-$\kappa$B pathway. Each method was applied to analyze this system, resulting in finding different subsets of important processes that might be prospective targets for changing this system behavior. While some of the processes were indicated in each of the approaches, others were found by one method only and would be missed if only that method was applied. This leads to the conclusion about the complementarity of the methods under investigation. The dual modeling approach of comprehensive structural and parametric analysis yields results that would not be possible if these two modeling approaches were applied separately. The combined approach, proposed in this paper, facilitates finding not only key processes, with which significant parameters are associated, but also significant modules, corresponding to subsystems of regulatory networks. The results provide broader insight into therapy targets in diseases in which the natural control of intracellular processes is disturbed, leading to the development of more effective therapies in medicine.

## Introduction

Ordinary differential equations (ODEs) and Petri nets are widely used in modeling and analysis of biological systems, from simple biochemical reactions to intracellular signalling pathways, regulatory networks, and complex biological systems^1–6^. Though usually research utilizes only one of these approaches, there are papers in which they are combined^7–9^. However, these studies generally employ continuous Petri nets^10^, which, among others, can be used as a basis for the automatic generation of ODEs.

This study proposes an alternative approach. We used an existing ODE model to create a classical Petri net model without any automatic conversion. Next, to perform complex analysis, we applied a significance analysis, focused on the model structure, for a classical Petri net model, and sensitivity analysis, focused on dynamics of model responses, for the corresponding ODE model. That led to finding processes being the most important for the complex system response, looking from two different perspectives. Our previous study showed that both methods lead to similar conclusions and may be treated as alternative approaches in such investigations. However, that preliminary conclusion was reached after analyzing a relatively simple biological model^11^. In this work, analysis of much more complex biological system shows that these approaches complement each other and employing both of them allows to extract more information from analysis than using a single one.

To illustrate advantages of using both ODE and Petri net models, an existing ODE model of DNA damage response system was chosen. It is based on the ATM/p53/NF-$\kappa$B pathways described in the work of Jonak et al.^12^. As it determines the cell fate, understanding its intricacies is very important from clinical perspective, for anti-cancer therapies. The model combines two key regulatory modules—p53 and NF-$\kappa$B, determining cell survival and its response to stress factors, with the activator module ATM, responsible for double-strand breaks (DSBs) detection. In this paper, Petri nets and ODEs-based approaches were used to perform a comprehensive analysis of that model, aimed at finding those processes, and kinetic parameters associated with them, that are the most important for system behavior. Once found, they should become the focus of much deeper experimental investigation as the best prospective targets in new drugs development.

Tumor protein p53 is a transcription factor, which, due to its main functions, is often called “the Guardian of the Genome”^13^. Its main role is to initiate DNA repair processes and stop the cell cycle following DNA damage, to prevent the division of the damaged cells. Another function, which is triggered when the DNA damage repair is either not possible or takes too much time, is initiation of the programmed cell death, called apoptosis^14,15^. p53 protein dysfunctions are exhibited in many types of cancer, promoting its progression. It has been shown that about half of the cancer types have mutations in the p53 gene, while in many others malfunctions of other proteins involved in the p53 signalling pathway are observed^16^.

Nuclear Factor $\kappa$B (NF-$\kappa$B) is a transcription factor mainly responsible for early immune response^17,18^. It is activated by a variety of types of stimuli, including proinflammatory cytokines, such as Tumour Necrosis Factor $\alpha$ (TNF$\alpha$) and Interleukin 1$\beta$ (IL1$\beta$), bacterial products, viruses, foreign DNA/RNA and many others^19^. NF-$\kappa$B regulates many processes, such as apoptosis, cell cycle progression, angiogenesis, and metastasis^20,21^. NF-$\kappa$B also plays a key role in cancer progression. Up-regulation of the NF-$\kappa$B pathway is frequently observed in cancer cells, which may contribute to their resistance to treatment^22^. The frequency and amplitude of NF-$\kappa$B oscillations was shown to control target gene expression^23–25^ and may have both proapoptotic or antiapoptotic functions^20^.

Numerous literature reports indicate that p53 and NF-$\kappa$B collectively control cell responses to stress, e.g. inducing apoptosis, cellular senescence or cell cycle arrest^19,26^. There are numerous pathways linking these two systems, with some interactions between p53 and NF-$\kappa$B being cooperative and some antagonistic. The most evident interaction can be seen at transcriptional level: NF-$\kappa$B upregulates the transcription of p53, whereas p53 attenuates transcription of NF-$\kappa$B inhibitors: I$\kappa$B$\alpha$ and A20^27^.

Another component of the system analyzed in this paper is Ataxia telangiectasia mutated (ATM) signaling pathway. It is responsible for DNA damage detection, especially DSBs, signal amplification and transmitting it to other regulatory modules such as p53 and NF-$\kappa$B^12^. Malfunctions of the detector module ATM are the most dangerous from the cell’s perspective, as they allow for passing the damaged DNA to the daughter cells. This may lead, among others, to premature aging^28^ or cancer^29^.

Taking into account that mathematical modeling of biological systems should support medicine and disease treatment^30^, and that the pathways mentioned above determine cell fate, they should become the focus of investigation as natural targets for any treatment that aims either at increasing apoptosis probability, following application of a killing agent, or increasing chances of cell recovery after environmental stress-induced DNA damage (see, e.g., Kozlowska and Puszynski^31^). However, the complex structure of involved regulatory networks makes it difficult to find signaling pathways components, whose change, affected by prospective drugs, would alter cell behavior to the greatest extent. Therefore, two distinct methods were applied: sensitivity analysis and significance analysis, each focused on a particular form of the model. The first method was employed to analyze the ODE model, that originally was published by Jonak et al.^12^. The ODE model structure was used to construct a Petri net model. Both models have two independent inputs that activate system responses, one representing TNF$\alpha$ treatment and another, representing ionizing radiation (IR).

Sensitivity analysis allows to identify the most important parameters in the ODE-model, change of which results in the greatest changes of the model responses. Since each ODE model parameter is related to a single biochemical process, this analysis can be used to search for molecular targets for drugs. Pharmacological altering of these processes should result in a strong therapeutic effect and thus lead to more effective therapies. On the other hand, the significance analysis of the Petri net allows the detection of both the most important subprocesses and the key modules. Influencing entire modules (not just individual processes) could potentially result in even better outcomes in disease treatment.

Results of both types of analysis were compared, to find out which conclusions overlap and which are different and what are their biological implications.

## Results

### Models

In this study, we analyze two models, describing the same signaling pathway but implemented using two different methods: ODEs and Petri nets. The ODEs model describing the mechanism of the DNA damage response system based on the ATM/p53/NF-$\kappa$B pathways^12^ was used as the basis for manual developing a corresponding model based on the Petri nets. Classical Petri nets enable creation of a qualitative model based on interactions between components, without any data on temporal system responses.

#### “Translation” of the ODE model into a Petri net

**Figure 1:**
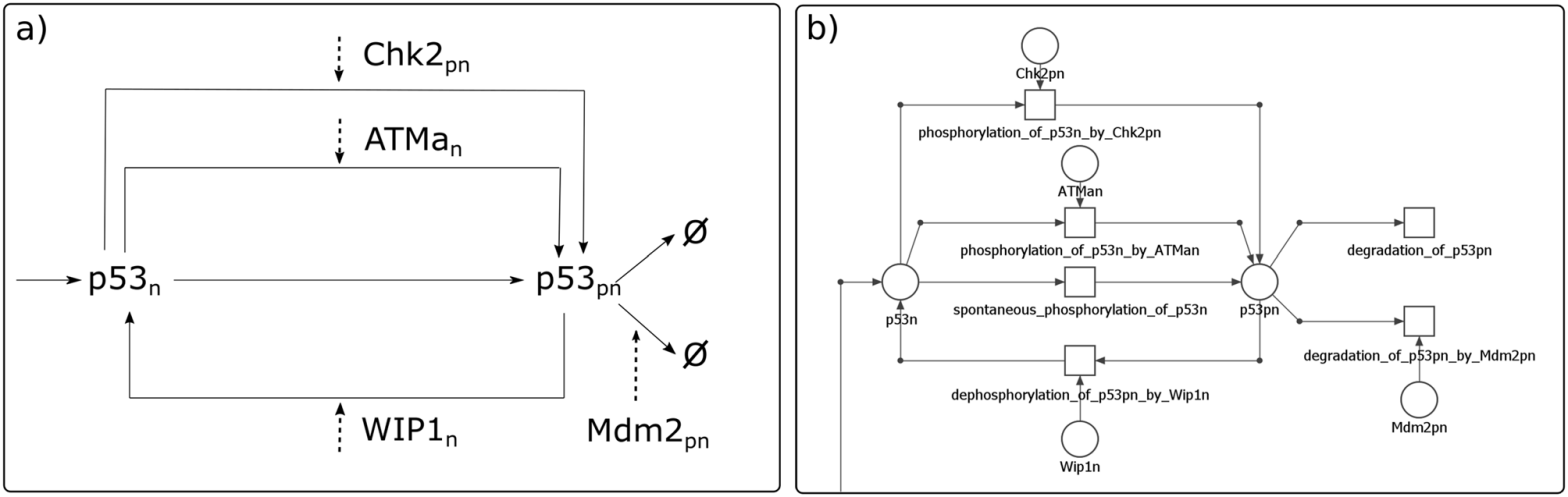
ODE-based wiring diagram vs. Petri net model.

Diagrams depicting biochemical reaction networks, whose kinetics is the basis for creation of ODE models, resemble, to some extent, Petri net model representation. However, transformation of the former into the latter is not necessarily straightforward. An example, illustrating a part of the network analyzed in this paper, is shown in Fig. 1a. In the figure, text blocks represent molecules taking part in the processes, such as synthesized transcripts, proteins and their complexes. Solid arrows represent reactions, including transition of molecules into a different (e.g., active) form. Dashed arrows indicate that the molecule given in the associated text box affects a reaction represented by the solid arrow. An arrow ending with a sharp point represents stimulation of a reaction by a given compound, while a blunt point means inhibition of a reaction. Figure 1b shows a corresponding Petri net model, where text blocks from ODE schemas were translated into places, and reactions with their regulatory mechanisms into transitions. It should be noted that a model based on a classical Petri net is qualitative. Therefore, translation of ODE model into this kind of Petri net involves retaining the structure of the model but quantitative information, such as values of kinetic reaction rates or algebraic expressions describing kinetic rates, is not taken into account. In classical Petri nets, different types of arcs are not distinguished, contrary to the arcs in the diagrams used for ODE models. In a model based on Petri nets different types of reactions, like the transition of a given compound into an active form, forcing a reaction, inhibition of a reaction or degradation are marked by a classic directed arc (for example, there are no inhibitor arcs). Due to these limitations, the proposed Petri net model had to be adapted, in particular to allow for including terms of inhibiting specific reactions, degradation as well as enzymatic reactions. The first limitation related to inhibition reactions and degradation was solved in the way described by Gutowska et al.^32^. This solution is based on creating an additional transition that prevents the firing of a selected reaction (i.e., transition) by taking tokens from its pre-places. That way, a specific reaction is weakened (inhibited). A complete inhibition of a reaction is realized through knockout analysis, described farther in the text. The second limitation is associated with enzymatic reactions. Enzymes that take part in a reaction should not be removed but should be available for next reaction. Therefore, pools of particular components have been created to ensure their return to circulation. For example, a transition $t_{158}$ corresponds in a biological context to the creation of a pool of PIP3.

To illustrate how the ODE model was manually translated into the Petri net model, let us consider one of its equations, describing change of the level of nuclear phosphorylated p53 protein ($p53_{pn}$), depicted in Fig. 1a:

1 $$\begin{aligned} \begin{aligned} \frac{d p53_{pn}}{dt}&= \left(pa_1 + pa_2 \frac{ATM_{an}(t) }{ATM_{an}(t) + pm_1} + pa_3 \frac{CHK2_{pn}(t)}{CHK2_{pn}(t) + pm_2}\right)p53_{n}(t)\\&\quad - pc_1P53_{pn}(t) WIP1_n(t) - (pd_4 + pd_5 MDM2_{pn} ^2 (t))p53_{pn}(t) \end{aligned} \end{aligned}$$

In Eq. (1), subsequent expressions describe, respectively (in the order in which they appear in the equation): spontaneous p53 activation, ATM-dependent and Chk2-dependent p53 activation, Wip1-dependent inactivation, spontaneous and Mdm2-dependent degradation. They correspond to particular fragments of the the Petri net (Fig. 1b):

- Spontaneous p53 activation. Three elements of the Petri net are needed for modeling this subprocess: a place (p53n) preceding a transition (spontaneous phosphorylation of p53n), the transition itself, and a place (p53pn) succeeding the transition (spontaneous phosphorylation of p53n). Due to the fact that it is a spontaneous reaction, no additional component (an additional preceding place) is needed to stimulate the reaction.
- ATM-dependent and Chk2-dependent p53 activation: Both reactions occur independently of each other, therefore they are modeled as two independent subprocesses. The following elements of the Petri net are required for modeling this subprocess: two places (p53n and ATM) preceding the transition (phosphorylation of p53n by ATM), the transition itself and a place (p53pn) succeeding the transition (phosphorylation of p53n by ATM). Two components are required to fire the transition (phosphorylation of p53n by ATM). Chk2-dependent p53 activation is realized in the same way.
- Wip1-dependent p53 inactivation: Activation/inactivation (phosphorylation/dephosphorylation) is modeled similarly. The only difference is the direction of arcs (activation: p53n $\rightarrow$ activation $\rightarrow$ p53pn, inactivation: p53n $\leftarrow$ inactivation $\leftarrow$ p53pn). The following elements of the Petri net are needed for modeling this subprocess: two places (p53pn and Wip1n) preceding transition (dephosphorylation of p53pn by Wip1n), this transition and place (p53n) succeeding the transition (dephosphorylation of p53pn by Wip1n).
- Spontaneous and Mdm2-dependent p53 degradation: A transition corresponding to degradation, unlike other reactions, does not have places succeeding it (such a transition can be treated as an output transition). Two elements of the Petri net are required for modeling of spontaneous degradation: a place (p53pn) preceding transition (degradation of p53pn), and the transition itself. In the case of Mdm2-dependent degradation two places (p53pn and Mdm2pn) preceding transition (degradation of p53pn by Mdm2pn), and this transition are needed. In this case, explicit output transitions are required to prevent further tokens flow. These output transitions, in addition to represent degradation, facilitate covering the Petri net model with t-invariants, which is a prerequisite for conducting the analysis.
- p53 production: Similarly, as in the case of the ODEs model (which is presented in Fig. 1a) as a wiring diagram), this subprocess is symbolically represented in Fig. 1b) by a directed arc.

The processes of inactive p53 protein production (gene activation, transcription and translation) are included in other equations, which is why they are marked symbolically to the left of the block diagram (Fig. 1a) as an arrow.

#### The ODE-based model

An extensive description of the ODE-based model used in this paper can be found in Jonak et al.^12^. In this model, ordinary differential equations were used to describe the dynamics of 61 components, such as proteins and transcripts, taking into account different possible forms of the same protein (e.g., phosphorylated and unphosphorylated). The model contains 135 parameters describing both the biochemical reaction rates and the physical properties of the simulated cell (e.g., the cytoplasmic to nuclear volume ratio or the number of alleles of a given gene). Simulations were run using ode23tb function in Matlab, with parameter values taken from Jonak et al.^12^.

Using the implemented model, it is possible to perform simulations with various combinations of input signals. The first input signal represents tumor necrosis factor $\alpha$ (abbreviated as TNF farther in the text), which stimulates the canonical NF-$\kappa$B signaling pathway. The second one represents ionizing radiation, which leads to DNA damage and subsequent activation of the p53 signaling pathway as well as NF-$\kappa$B signaling pathway.

We defined two simulation protocols for further analysis. These protocols differ only in the total irradiation dose. In each protocol, simulations were divided into three consecutive phases:

1. Waiting for equilibrium phase—24 h phase during which the model reaches steady state (no IR, no TNF).
2. Stimulation phase—the phase in which signaling pathways are stimulated by the simultaneous activation of TNF (10 ng/ml) and IR (1 h irradiation with a total dose of 4 or 10 Gy).
3. After-irradiation phase—240 h phase during which the cell attempts to repair DNA damage induced in phase 2 (no IR, TNF still present at 10 ng/ml).

**Figure 2:**
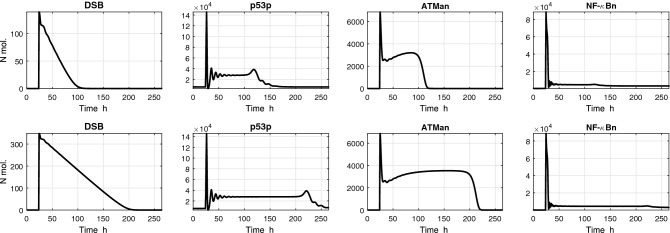
Time courses of selected variables in the the ATM/p53/NF-$\kappa$B signaling pathway model described in Jonak et al.^12^. Simulation was performed with two input signals: (TOP PANELS) 10 ng/ml TNF and 4 Gy IR; (BOTTOM PANELS) 10 ng/ml TNF and 10 Gy IR. TNF stimulation is constant during the simulation, IR radiation is turned on after 24 h simulation for 1 h.

Sample time courses corresponding to these simulation protocols are shown in Fig. 2.

#### Model based on Petri nets

**Figure 3:**
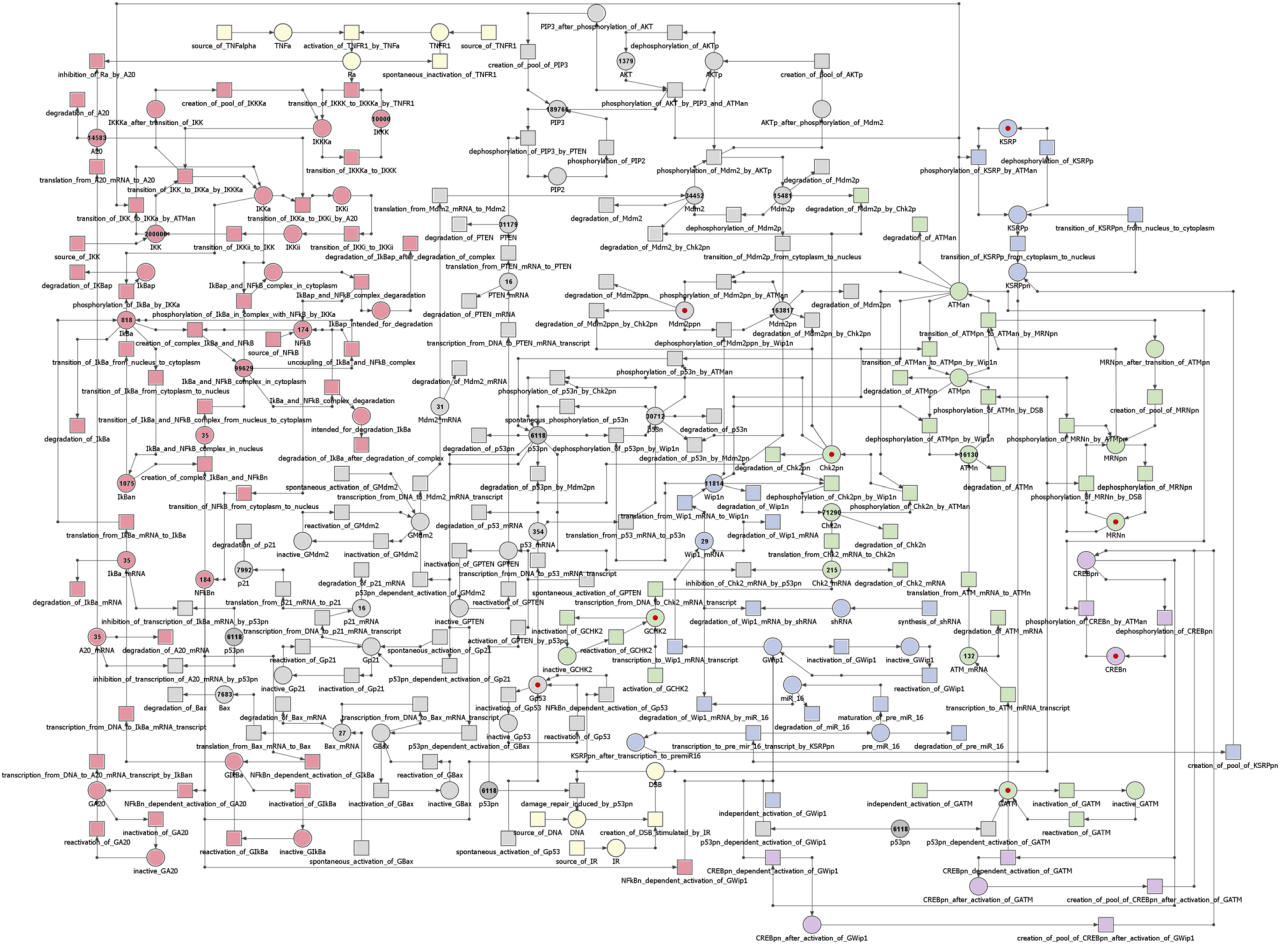
The proposed model of the ATM–p53–NF-$\kappa$B pathways with two excitations (TNF and IR) (541 t-inv). The presented Petri net is divided into several modules: ATM (green), p53 (gray), NF-$\kappa$B (red), WIP1 (blue), CREB (purple) and inputs of the system (yellow). The places which are marked with the same color and name correspond to the same particle (logic place), they are used only for transparency in the model.

The proposed model concentrate on ATM, p53, NF-$\kappa$B pathways, and excitations associated with the presence of TNF and IR. This model contains 170 transitions (elementary processes) and 89 places (biological components). Furthermore, it is covered by 541 t-invariants. All place and transition names are listed in Supplementary Table S1 and S2 (available online in Supplementary information section). Figure 3 shows the proposed model, taking into account division into particular modules: ATM (green), p53 (gray), NF-$\kappa$B (red), WIP1 (blue), CREB (purple) and extortions of the system (yellow). The Petri net model was created using a tool called Holmes^33^, facilitating manual creation of this type of models. The described model is available for download in two different formats, using links given in the section Supplementary information. The first one, .project, is a Holmes-specific format, while the second one, .spped, is a format specific to Snoopy (another tool used to model Petri nets)^34^. Holmes supports both of these formats, and the manual for Holmes software is available at the link given by Radom et al.^33^.

Adequacy of Petri net model and ODE model was confirmed by comparing crucial checkpoints, obtained for different inputs setup. For that aim, knockouts of modules associated with particular inputs were used, i.e., the knockout of a single input module (TNF or IR) and the knockout of both input modules (TNF and IR). The latter one corresponds to a steady state. That way, the system response to a TNF activation can be analyzed by a knockout analysis of the IR module. Similarly, the system response to an IR activation can be analyzed by a knockout analysis of the TNF module. In the case of TNF activation a significant activation of the NF-$\kappa$B pathway, and modules closely associated with it, should be expected, which is confirmed by the results from a knockout of IR module in the proposed model. For IR excitation, significant activation of p53 pathway is observed experimentally. Such behavior is also visible in knockout of TNF module in the Petri net model. Furthermore, homeostasis (without any of the TNF or IR inputs) is characterized by low levels of Chk2, ATM, p53p, and very low levels of p21 and BAX. Corresponding knockouts of TNF and IR modules in the proposed model yield such results.

### Sensitivity analysis of ODE-based model

In this paper, we used the one-at-a-time (OAT) method based on the frequency distribution of a model transient response described in the work of Kardynska and Smieja^35^. Following the procedure proposed in Kardynska and Smieja^35^ numerous simulations with randomly changed parameters were carried out. For each parameter in the model, 1000 simulations were carried out in which the value of this parameter was randomized within the range of $\pm \,30\%$ of its nominal value using a uniform distribution. Only the kinetic parameters of the model (such as the rates of biochemical reactions) were analyzed, as only these may become possible molecular targets for drugs. Moreover, only the parameters related to specific biochemical reactions have their counterparts in the Petri net as transitions. As a result, the number of analyzed parameters was reduced to 123 (from the initial 135). These parameters are listed in Supplementary Table S4.

The algorithm described in the work of Kardynska and Smieja^35^ suggests selecting one model variable (e.g., the concentration of one of the proteins present in the model) as a representation of the model response. In the case of the model ATM/p53/NF-$\kappa$B, which combined multiple signaling pathways, finding such a single variable proved very difficult. To solve this problem, we decided to take a different approach and define a combined model response, based on time courses of all variables in the model. First, we created parameter rankings for each model variable according to the procedure described in Kardynska and Smieja^35^. Then we calculated the arithmetic mean of the sensitivity indices for parameters from all ranking (for all model variables). The resulting parameter ranking represents the cumulative effect of changing a given parameter value on all variables in the model. It should be noted that a high position of a given parameter in the ranking may suggest that a change of this parameter value results in a substantial change in few model variables or a moderate change in many model variables. Parameters ranking does not make distinguishing between the two cases possible.

**Figure 4:**
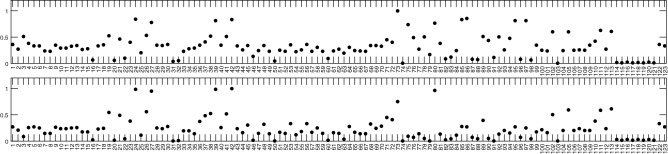
Parameters ranking for the ATM/p53/NF-$\kappa$B signaling pathway model described in Jonak et al.^12^. Simulation was performed with two input signals: (TOP PANEL) 10 ng/ml TNF and 4 Gy IR; (BOTTOM PANEL) 10 ng/ml TNF and 10 Gy IR. Parameter names corresponding to the numbers on the ranking are given in Supplementary Table S4.

We found that many parameters substantially affect the model response (Fig. 4). Considering the structure of the model, which combines several signaling pathways, results were discussed separately for groups of parameters associated with the p53 and NF-$\kappa$B module, the DNA damage detection module (related to ATM protein), Wip1/KSRP module and parameters related to the process of cell cycle arrest and apoptosis (Bax/p21 proteins).

#### Analysis of 10 ng/ml TNF and 4 Gy IR simulation results

In the simulation with a low dose of radiation, sensitivity analysis showed that the parameter with the greatest impact on the model response is a DSB damage rate caused by IR. That finding is not surprising. Unfortunately, it does not lead to any conclusions concerning prospective molecular drug targets.

Among the parameters of the p53 signaling module, many are particularly important, including those directly affecting the level of p53 protein (synthesis of p53 mRNA, p53 transcript degradation, p53 translation rate) and its inhibitor, Mdm2 (synthesis of Mdm2 mRNA, Mdm2 transcript degradation, Mdm2 translation rate, Mdm2p protein spontaneous degradation, Mdm2 degradation by Chk2).

Surprisingly, the entire NF-$\kappa$B signaling module turned out to be very resistant to changes in parameters (compared to the remaining signaling pathways combined in the analyzed model) and none of its parameters substantially affects the model response. This may result from the fact that the NF-$\kappa$B responds to the TNF or IR very quickly, and its response is also quickly suppressed, resulting in low levels of the proteins involved. Unlike them, proteins associated with the p53 signaling pathway persist at elevated levels for several days following pathway excitation.

Contrary to NF-$\kappa$B pathway, the DNA damage detection module was very sensitive and many of its parameters strongly influenced the response of the entire model. Within parameters related to the ATM module, parameters representing the rates of DNA damage creation (DSB damage rate caused by IR) and repair (DSB repair rate and Michaelis-Menten constant for DSB repair) have proved to be extremely important. Parameters associated with ATM gene activation, ATM transcription and synthesis as well as degradation rates for both ATM protein and ATM transcript also seem to have a great impact on the model response. Other relevant parameters include ATM activation rate by DSB formation and ATM activation rate by MRN complex formation; Chk2 gene activation and inactivation rates; MRN complex activation rate by ATM.

Of the parameters describing the dynamics of the Wip1/KSRP module only those related to WIP1 mRNA synthesis and spontaneous degradation, as well as WIP1 translation and Wip1 protein degradation turned out to be important.

The analysis showed a very low influence of the parameters related to the dynamics of Bax and p21 proteins. Bax and p21 proteins do not affect other processes described in the model; instead, they can be considered as output signals of the model, enabling the cell cycle arrest and subsequent activation of the apoptosis process, if necessary. However, these processes are not described in the analyzed model.

#### Analysis of 10 ng/ml TNF and 10 Gy IR simulation results

In the simulation with a high dose of radiation, sensitivity analysis showed that the parameter with the greatest impact on the model response is Mdm2 translation rate. This parameter appeared in the previous analysis but was not the most important one. In fact, all parameters mentioned in the previous subsection proved to significantly affect the model response in the simulation with 10 Gy. In addition to them, parameters of processes forming a positive feedback loop in the p53 control system (Mdm2 and PTEN gene inactivation) and p53 degradation by Mdm2 were crucial for the model response.

Substantial differences in the parameter rankings were observed for the ATM module. The only two parameters found to be relevant to the model response in the simulation with a high dose of radiation are the rates of DNA damage creation (DSB damage rate caused by IR) and repair (DSB repair rate).

No changes in the significance of the parameters for modules NF-$\kappa$B, Wip1/KSRP, Bax and p21 have been observed.

### Analysis of Petri net-based model

The analysis for the model based on Petri nets is based on significance analysis at the level of individual transitions and significance analysis at the level of transition subsets (searching for a certain set of transitions that is included in a sufficiently large number of t-invariant supports).

The significance analysis finds subprocesses that are the most crucial for the functioning of the system under investigation, i.e. subprocesses leading to DNA damage and repair processes. The results showed that the most important elementary processes in the proposed model are associated with creation of DSB by IR, ATM, p53, and NF-$\kappa$B. Creation of DSB by IR is involved in over 77% of all modeled subprocesses. ATM and p53 subprocesses are involved in over 75% of all modeled subprocesses, while the NF-$\kappa$B module is involved in over 47% of them. The exact values for individual elementary processes are provided in Table 1, which contains only selected most relevant reactions. The results of the significance analysis for the remaining reactions (transitions) are given in Supplementary Table S3.

**Table 1:** Significance analysis for the proposed Petri net model with two excitations (541 t-inv).

Significance analysis			
Model of ATM-p53-NF-$\kappa$B with presence of TNF and IR (541 t-inv)			
No.	Name of transition	t-inv	Frequency trans/t-inv (%)
$t_{1}$	Creation of DSB stimulated by IR	418	77.26
$t_{2}$	Source of IR	418	77.26
$t_{42}$	Transcription to ATM mRNA transcript	418	77.26
$t_{13}$	Source of DNA	416	76.89
$t_{14}$	Transcription from DNA to p53 mRNA transcript	416	76.89
$t_{52}$	Phosphorylation of ATMn by DSB	415	76.71
$t_{16}$	Translation from p53 mRNA to p53n	414	76.52
$t_{43}$	Translation from ATM mRNA to ATMn	414	76.52
$t_{54}$	Transition of ATMpn to ATMan by MRNpn	407	75.23
$t_{162}$	Creation of pool of MRNpn	407	75.23
$t_{107}$	Transition of NF-$\kappa$B from cytoplasm to nucleus	259	47.87
$t_{145}$	Source of NF-$\kappa$B	258	47.69
$t_{39}$	Phosphorylation of Chk2n by ATMan	222	41.04

Structural analysis also includes searching for a set of transitions that is included in a sufficiently large number of t-invariant supports. To find that set, we used an algorithm described in the work by Gutowska and Formanowicz^36^. The result of this algorithm for the basic version of the model (541 t-invariants) allowed the determination of significant subprocess, which consists of the following transitions: $\hbox {t}_{{1}}$, $\hbox {t}_{{2}}$, $\hbox {t}_{{13}}$, $\hbox {t}_{{14}}$, $\hbox {t}_{{16}}$, $\hbox {t}_{{42}}$, $\hbox {t}_{{43}}$, $\hbox {t}_{{52}}$, $\hbox {t}_{{70}}$, $\hbox {t}_{{107}}$, $\hbox {t}_{{147}}$ (the names of the listed transitions are included in Supplementary Table S2). From biological perspective, such subset indicates key elements in the mechanism of the DNA damage response system based on ATM/p53/NF-$\kappa$B pathways. After the DNA damage, ATM detection module activates p53 and NF-$\kappa$B, which cooperate to determine the cell’s response to the stimulus. Knockout of such subsets leads to exclusion of about 92% of all modeled subprocesses (what clearly emphasizes its importance).

## Discussion

The independent analysis of the Petri net-based model, as well as the independent analysis of the ODE model, indicate significant components in the proposed model. In the case of the Petri net-based model analysis, we get information about crucial elementary processes (reactions), although we can also obtain such information for subprocesses and entire modules. In the case of ODE analysis, we obtain information about the parameters that have the greatest impact on the model response.

The significance analysis of the Petri net-based model is a structural analysis and allows indicating essential subprocesses in terms of information flow in such a net. While, the sensitivity analysis of the ODE-based model allows indicating the parameters whose change results in the greatest quantitative and qualitative changes in the model response. The results of the sensitivity analysis are influenced by both the values of parameters and inputs, therefore the analysis was performed for different simulation conditions.

Some of the results for the significance and the sensitivity analysis are consistent (especially for the 4Gy dose; see Table 2). However, each method provides additional conclusions that another one is not able to yield. Therefore, their results indicate that the methods are complementary, in particular, in view of the following observations:

- One of the most important elementary processes for the Petri net model concern ATM module and corresponding to them transitions $t_{42}$, $t_{43}$, $t_{52}$, $t_{54}$ (see Supplementary Table S2)—from transcription, through translation, to ATM phosphorylation. This module is a key module for damage detection in response to IR radiation (it activates p53 and NF-$\kappa$B pathways). The parameters related to ATM module turned out to be important also in the ODE sensitivity analysis (parameters $p_{75}$, $p_{76}$, $p_{95}$, $_{97}$ in the ODE model (see Supplementary Table S4)), but only for the simulation with 4Gy irradiation. Under extreme conditions (10Gy), the significance of these parameters is lower, which may be related to the existence of a strong, positive feedback loop in this regulatory system.
- The sensitivity analysis showed a significant influence of parameters related to repair of double-stranded DNA damage (DSB repair), degradation of proteins, and degradation of ATM and Mdm2 transcripts. These elementary processes were not detected in the significance analysis for the Petri net model.

**Table 2:** Comparison of results of SA of ODE model (simulations with input signals (**A**) 10 $\upmu$M TNF and 4 Gy IR and (**B**) 10 $\upmu$M TNF and 10 Gy IR) and analysis of Petri net model based on the selected parameters with the most significant impact on the modeled system.

A*				
Biological process	Sensitivity analysis of ODE-based model		Significance analysis of Petri net-based model	
	Parameter no.	Pos. in ranking	Transition no.	frequency trans/t-inv (%)
IR dependent DSB formation	73	1	**t1**	**77.26**
Degradation of ATMn/ATMpn/ATMan	86	2	t44/53/56	0.74
Degradation of Mdm2 mRNA	24	3	t19	2.03
Translation of Mdm2	42	4	**t31**	**28.65**
Degradation of ATM mRNA	85	5	t45	0.74
Transcription of ATM	97	6	**t42**	**77.26**
Transcription of Mdm2	39	7	**t17**	**30.68**
Translation of ATM	95	8	**t43**	**76.52**
Degradation of Mdm2p induced by Chk2p	27	9	t29	3.32
DNA damage repair induced by p53	80	10	t0	1.84
B*				
Translation of Mdm2	42	1	**t31**	**28.65**
Transcription of Mdm2	39	2	**t17**	**30.68**
Degradation of Mdm2 mRNA	24	3	t19	2.03
DNA damage repair induced by p53	80	4	t0	1.84
Degradation of Mdm2p induced by Chk2p	27	5	t29	3.32
IR dependent DSB formation	73	6	**t1**	**77.26**
Ttranslation of Wip1	11	7	**t32**	**21.44**
Degradation of Wip1 mRNA	10	8	t34	2.40
Transcription of Wip1	11	9	**t110**	**28.65**
Degradation of Mdm2p	26	10	t99	2.03

$^{*}$Transitions with significance above 20% are marked in bold font. It can be noticed that 50% of the results are common for ODE and Petri net models analyses, when comparing the significant reactions from the Petri net model to the significant parameters from the ODE model.

The fact that degradation reactions have not been detected as essential may be associated with the structure of the Petri net model. In that framework, the larger number of subprocesses is dependent on a reaction, the more important this reaction is. A transition corresponding to degradation (called an output transition) does not affect other reactions (does not have places succeeding it) in any way other than limiting the amount of molecules being degraded (by taking tokens from a given place). The significance analysis revealed other important subprocesses, mainly related to transcription and translation.

In the case of DNA damage repair (parameter $p_{80}$ in the ODE model and corresponding transition $t_{0}$ in the Petri net model), the structure of the Petri net model also influences significance of this transition. If all repair modules were directly associated with this particular transition, its significance would be greater. In the analyzed model, damage repair is induced by p53pn, which is engaged in many other subprocesses. Therefore, though most transitions associated indirectly with DNA repair were found in the Petri net model to be important, the specific transition corresponding to damage repair by p53pn was not.

**Figure 5:**
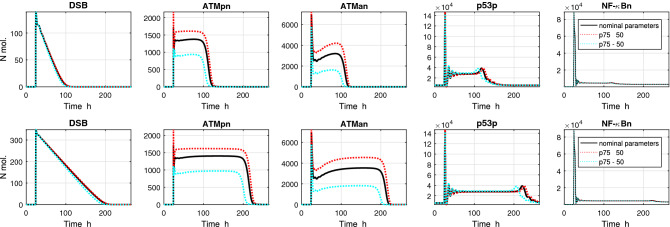
Impact of increasing/decreasing the value of the parameter $ma_3$ (par. No. 75) on the model’s response. Simulation was performed with two input signals: (TOP PANELS) 10 ng/ml TNF and 4 Gy IR; (BOTTOM PANELS) 10 ng/ml TNF and 10 Gy IR. TNF stimulation is constant during the simulation, IR radiation is turned on after 24 h simulation for 1 h.

**Figure 6:**
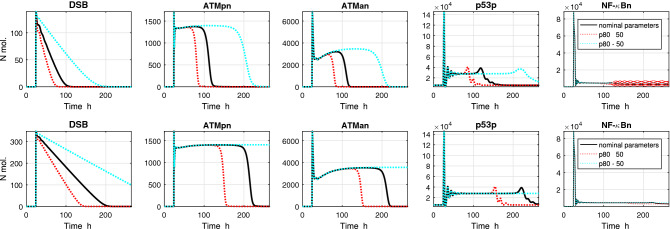
Impact of increasing/decreasing the value of the parameter $mc_1$ (par. No. 80) on the model’s response. Simulation was performed with two input signals: (TOP PANELS) 10 ng/ml TNF and 4 Gy IR; (BOTTOM PANELS) 10 ng/ml TNF and 10 Gy IR. TNF stimulation is constant during the simulation, IR radiation is turned on after 24 h simulation for 1 h.

Interestingly, though in ODE model simulation for varying parameter $p_{75}$ (indicated as significant in Petri net model analysis, and less significant in ODE analysis), differences in the levels of the various forms of the ATM protein were noticed, no differences in settling time of the response were observed (see Fig. 5). Contrary to parameter $p_{75}$, changes in parameter $p_{80}$ (indicated as not significant in Petri net model analysis, and significant in ODE analysis) do not result in differences in protein levels but result in different settling time of the response, see Fig. 6. Whether this is a coincidence, or a general rule, and how to explain these relationships remains an open question.

## Conclusion

There are various methods of modeling complex biological systems, but each has its own limitations. Therefore, in this work, a dual approach, consisting in using two different modeling methods has been proposed. It facilitates a comprehensive structural and simulation analysis. Complementarity of sensitivity analysis (for ODEs model) and significance analysis (for Petri net model) has been confirmed by the results obtained for a sample large-scale model of DNA damage response system based on the ATM/p53/NF-$\kappa$B pathways. Conclusions reached by application of only one of the methods would miss important information provided by another method, as described in sections “Results”.

An important, though unexpected finding is that changing parameter indicated as significant in the Petri net and less significant in ODE model leads to changing in protein levels, without changing of the settling time. On the other hand, changing a parameter rendered important in the ODE model, which is less important in the Petri net, leads to an opposite conclusion.

Our research shows that conducting a comprehensive analysis, both structural (for the Petri nets model) and parametric (for the ODE model), may not only allow for a better understanding of the studied phenomenon, but also allows to find essential parameters and significant modules which may prove important for the development of new, more effective therapies. Therefore, we show that the dual modeling approach can better supports the goals of medical therapy, and in the context of ATM/p53/NF-$\kappa$B pathways, it may lead to better outcomes in cancer therapy.

## Methods

### ODE-based approach

When the mathematical modeling of the intracellular signaling pathways is considered, usually one of the two main approaches is implemented: deterministic through ODEs or stochastic using Gillespie algorithm. Since parametric sensitivity analysis methods for the first type of models are well established, an ODE model is considered in this paper.

ODE models describe changes of concentration, or number of molecules, of molecular players, such as proteins, mRNAs, miRNAs, protein complexes etc., involved in the processes under investigation. They require parameter estimation which is based on the experimental results. Each process, taken into account, is represented by a single algebraic expression that appears in one or more equations and contains one or several parameters. A single parameter is associated with only one process. Therefore, high sensitivity of the model with respect to a given parameter indicates that system responses might be significantly altered by targeting the associated process. That, in turn, might be achieved by finding a molecule that would bind to a molecular player involved in this process, thus linking sensitivity analysis to search for molecular drug targets. Sensitivity analysis is the most important one taking into account the searching for the possible therapeutic target.

Sensitivity analysis became one of the necessary tools in in silico investigation of signaling pathways, as it provides information not only about dependence between parameter values and system behavior, but also about robustness of these systems^37^, a property that should characterize most of the pathways. This property is related to many biological phenomena such as homeostasis, stability, redundancy and plasticity^38^. SA methods can serve a number of useful purposes, e.g., uncover technical errors in the model, identify critical regions in the parameter space or establish priorities for research^39^. Among others, they also provide means to simplify high dimensional models that arise in systems biology^40^ and can be used to indicate prospective molecular targets for new drugs against diseases associated with particular signaling pathways^41^.

Various methods of SA have been proposed, developed for either local or global sensitivity of systems under investigation. The local sensitivity analysis provides information about the effect of a small deviation of a single parameter from its nominal value on the system output. Global sensitivities, in turn, describe how the system output changes when multiple parameters change within a relatively wide range. Local methods are usually based on determining derivatives of model outputs with respect to parameters. Their undoubted advantage is simplicity and low computing power needed to perform the analysis, however, these methods do not guarantee correct results in the case of strongly non-linear models^39^. Global methods based on variance do not have such limitations, however, in the case of complex models of signaling pathways, they require enormous computing power, which may be a factor limiting their use. The compromise here may be variance-based methods from the OAT group, in which single parameters are randomly changed within a relatively wide range.

### Petri net-based approach

Petri nets are mathematical objects with a structure of a directed weighted bipartite graph. A set of vertices of such a graph can be divided into two disjoint subsets in such a way that vertices belonging to the same subset are not connected by an arc. In the context of Petri nets, vertices being elements of one of these subsets are called places while elements of the other one are transitions. When a Petri net is a model of some system, places correspond to its elementary passive components (e.g., chemical compounds) and transitions are counterparts of elementary active components (e.g., chemical reactions). The bipartite graph describes the structure of the modeled system, but a Petri net is not just a graph. There is another type of component of such a net, i.e., tokens which bring a kind of dynamics into a net. They are located in places and flow from one place to another via transitions. This flow corresponds to a flow of substances, signals etc. through the modeled system. It is governed by the rule of firing transitions. According to this rule a transition is active if the number of tokens in all pre-places of this transition (i.e., the places which are its immediate predecessors) are equal to or greater than the weights of arcs connecting these places with the transition. An active transition can be fired what means that tokens can flow from its pre-places to its post-places (i.e., the places which are immediate successors of the transition). The number of flowing tokens is equal to the weights of the respective arcs.

Petri nets have a very intuitive graphical representation being helpful in understanding the structure and behavior of the modeled system. In this representation places are depicted as circles, transitions as rectangles, arcs as arrows and tokens as dots or numbers located in places. The weights, being positive integers, are represented as numbers associated with arcs (if a weight is equal to one, it is not shown).

Although the graphical representation of Petri nets is intuitive, it is not very well suited for a formal analysis of the proposed model. The used methods for analysis, are based on t-invariants, that is why for this purpose another representation, i.e., an incidence matrix, is used. In such matrix $A=[a_{ij}]_{n\times m}$, where *n* is the number of places and *m* is the number of transitions, rows correspond to places while columns correspond to transitions. Entry $a_{ij}$, being an integer number, is equal to a difference between the numbers of tokens located in place $p_i$ after and before firing transition $t_j$.

On the basis of matrix *A* invariants, which are especially important in the analysis of models of biological systems, can be calculated. There are two types of them, i.e., transition invariants (t-invariants) and place invariants (p-invariants). The former ones are vectors $x\in {\mathbb {N}}^m$ being solutions of equation $A\cdot x=0$, while the latter ones are vectors $y\in {\mathbb {N}}^n$ satisfying equation $y\cdot A=0$.

In the analysis presented in this paper t-invariants are used. With every such an invariant a set of transitions, called its support, is associated. Support *s*(*x*) of t-invariant *x* contains those transitions which correspond to positive entries in vector *x*, i.e., $s(x)=\{t_j: x_j>0, j=1,2,\ldots ,m\}$. If every transition $t_j$ from a support of t-invariant *x* is fired $x_j$ times, then the distribution of tokens over places of the whole net (also called a marking of the net) will not change, what means that the state of the modeled system is unchanged. Because a marking of a net corresponds to a state of the modeled system, it means that t-invariants correspond to subprocesses which do not change the state of the analyzed system and are crucial in the analysis of the Petri net-based models.

Methods of analysis of models based on Petri nets allow conducting structural analysis which includes significance analysis, knockout analysis, and searching for a significant subset of transitions. Such analyses are important to determine the significance of elementary processes or larger subprocesses.

The significance analysis is based on the frequency occurrence of each transition (elementary subprocess) in all supports of t-invariants^42^. On the basis of such frequency, the significance of transition expressed in percentage can be calculated, i.e., $\frac{f \cdot 100}{s} = S$ $[\%]$, where $f =$ frequency occurrence of transition, $s =$ all supports of t-invariants, and $S =$ significance of transition $[\%]$. Such an analysis allows to distinguish which subprocesses are more crucial for the functioning of the modeled system. Apart from determining the most significant elementary processes, it can be determined also the irrelevant ones. To complement the significance analysis a knockout analysis was performed (cf.^42^).

The knockout analysis allows to estimate which subprocesses have been excluded in consequence of knockout of selected transition or sets of transitions. It allows to estimate how crucial selected elementary processes or whole subprocesses (sets of elementary processes) are, based on the number of excluded subprocesses. In such analysis, it is necessary to determine the number of t-invariants before and after knockout. Using these numbers of t-invariants, it is possible to estimate the percentage of excluded t-invariants as a result of a knockout and on this basis conclude about the significance of the knockouted elementary process or subprocess, i.e., $\frac{(tinv_{b} - tinv_{a}) \cdot 100}{tinv_{b}} = E [\%]$, where $tinv_{b} =$ number of all t-invariants before knockout, $tinv_{a} =$ number of all t-invariants after knockout, and $E =$ percentage of excluded t-invariants $[\%]$.

An additional element of structural analysis is searching for a significant set of transitions. This approach is focused on finding a certain set of transitions that would be included in a sufficiently large number of t-invariant supports. We can distinguish two situations. The first one, a situation in which a certain set is included in almost all t-invariant supports (the greater number of t-invariant supports that include the searched set, the more important it is). Regardless of the expert knowledge about a given biological phenomenon, if a certain set occurs in more than 80% of all modeled subprocesses (supports of t-invariant), it will be considered as necessary for the system’s functioning. A separate issue concerns the size of such a set. The smaller the set, the greater chances that it will be included in a bigger number of t-invariant supports, therefore, the larger sets of transitions (that can have assigned biological significance) are of interest. In the second situation, a set can occur in a smaller number of t-invariant supports, but if these t-invariants are crucial for the functioning of the model, then finding such set of transitions is also valuable. However, this case, unlike the first one, requires expert knowledge at every level of analysis. It should be realised which subprocesses are crucial for the functioning of the system in accordance with the state of biological knowledge. In both cases, a knockout of such a set of transitions results in turning off many or even the most of the modeled subprocesses (t-invariants). Transitions that are being elements of such a subset correspond to certain elementary processes. These elementary processes occur in many subprocesses, which may turn out to be essential to the functioning of a modeled system.

## Supplementary Information

Supplementary Information.
