# Supplementary material for: Petri nets and ODEs as complementary methods for comprehensive analysis on an example of the ATM–p53–NF-κB signaling pathways
Source: Sci Rep. 2022 Jan 21;12:1135. doi: 10.1038/s41598-022-04849-0 (PMC8782877; doi:10.1038/s41598-022-04849-0)
Supplement: Supplementary file 1 — Supplementary Information. [file 41598_2022_4849_MOESM1_ESM.pdf]

Petri nets and ODEs as complementary methods for comprehensive analysis on an example of the ATM–p53–NF-κB signaling pathways

Kaja Gutowska[1,*,†], Daria Kogut[3,*,†], Malgorzata Kardynska[2,3,†], Piotr Formanowicz[1,4], Jaroslaw Smieja[3], and Krzysztof Puszynski[3]

[1]Institute of Computing Science, Poznan University of Technology, Poznan 60-965, Poland
[2]Department of Biosensors and Processing of Biomedical Signals, Silesian University of Technology, Zabrze 41-800, Poland
[3]Department of Systems Biology and Engineering, Silesian University of Technology, Gliwice 44-100, Poland
[4]Institute of Bioorganic Chemistry, Polish Academy of Sciences, Poznan 61-704, Poland

[*]To whom correspondence should be addressed.
[†]These authors contributed equally to this work

Abstract

This supplementary document provides additional details of the models and analysis, i.e., a description of the components of the Petri net-based model (list of places and transitions), the list of the parameters of the ODE-based model, the extended results of the significance analysis (extended for all transitions from the Petri net-based model), as well as information about the ODE model simulations and analysis.

Supporting information

Supplementary Table S1. List of places for the proposed Petri net model (541 t-inv).

No.	Biological meaning	No.	Biological meaning
p_0	DNA	p_{45}	IκBαn
p_1	DSB	p_{46}	IKKa
p_2	IR	p_{47}	IKKi
p_3	p21 mRNA	p_{48}	IKKii
p_4	Bax mRNA	p_{49}	IKK
p_5	p21	p_{50}	NF-κBn
p_6	Bax	p_{51}	IκBα
p_7	p53pn	p_{52}	IκBα and NF-κB complex in nucleus
p_8	p53n	p_{53}	IκBα and NF-κB complex in cytoplasm
p_9	p53 mRNA	p_{54}	NF-κB
p_{10}	Mdm2 mRNA	p_{55}	intended for degradation IκBα
p_{11}	PTEN mRNA	p_{56}	shRNA
p_{12}	PTEN	p_{57}	IκBαp
p_{13}	PIP3	p_{58}	IκBαp and NF-κB complex in cytoplasm
p_{14}	PIP2	p_{59}	IκBαp intended for degradation
p_{15}	AKT	p_{60}	GATM
p_{16}	AKTp	p_{61}	GCHK2
p_{17}	Mdm2	p_{62}	GPTEN
p_{18}	Mdm2p	p_{63}	GMdm2
p_{19}	Wip1 mRNA	p_{64}	Gp21
p_{20}	Wip1n	p_{65}	GBax
p_{21}	Chk2 mRNA	p_{66}	GA20

No.	Biological meaning	No.	Biological meaning
p_{22}	Chk2n	p_{67}	GIκBα
p_{23}	Chk2pn	p_{68}	GWip1
p_{24}	ATM mRNA	p_{69}	Gp53
p_{25}	ATMn	p_{70}	inactive GA20
p_{26}	pre miR 16	p_{71}	inactive GATM
p_{27}	miR 16	p_{72}	inactive GBax
p_{28}	ATMpn	p_{73}	inactive GCHK2
p_{29}	ATMan	p_{74}	inactive GIκBα
p_{30}	CREBn	p_{75}	inactive GMdm2
p_{31}	MRNn	p_{76}	inactive Gp21
p_{32}	MRNpn	p_{77}	inactive Gp53
p_{33}	KSRP	p_{78}	inactive GPTEN
p_{34}	KSRPpn	p_{79}	inactive GWip1
p_{35}	Mdm2pn	p_{80}	CREBpn
p_{36}	Mdm2ppn	p_{81}	KSRPp
p_{37}	TNFα	p_{82}	PIP3 after phosphorylation of AKT
p_{38}	TNFR1	p_{83}	AKTp after phosphorylation of Mdm2
p_{39}	Ra	p_{84}	KSRPpn after transcription to pre-miR16
p_{40}	IKKKa	p_{85}	CREBpn after activation of GATM
p_{41}	IKKK	p_{86}	CREBpn after activation of GWip1
p_{42}	A20	p_{87}	MRNpn after transition of ATMpn
p_{43}	A20 mRNA	p_{88}	IKKKa after transition of IKK
p_{44}	IκBα mRNA		

Supplementary Table S2. List of transitions for the proposed Petri net model (541 t-inv).

No.	Biological meaning	No.	Biological meaning
t_0	damage repair induced by p53pn	t_{85}	transition of IKKi to IKKii
t_1	creation of DSB stimulated by IR	t_{86}	transition of IKKii to IKK
t_2	source of IR	t_{87}	transition of IKK to IKKa by IKKKa
t_3	transcription from DNA to Bax mRNA transcript	t_{88}	inhibition of Ra by A20
t_4	transcription from DNA to p21 mRNA transcript	t_{89}	transition of IKK to IKKa by ATMan
t_5	translation from p21 mRNA to p21	t_{90}	inhibition of transcription of IκBα mRNA by p53pn
t_6	translation from Bax mRNA to Bax	t_{91}	inhibition of transcription of A20 mRNA by p53pn
t_7	degradation of p21	t_{92}	degradation of p21 mRNA
t_8	degradation of Bax	t_{93}	degradation of Bax mRNA
t_9	dephosphorylation of p53pn by Wip1n	t_{94}	degradation of p53pn
t_{10}	phosphorylation of p53n by Chk2pn	t_{95}	degradation of p53n
t_{11}	degradation of p53pn by Mdm2pn	t_{96}	degradation of Mdm2ppn
t_{12}	degradation of p53n by Mdm2pn	t_{97}	degradation of Mdm2pn
t_{13}	source of DNA	t_{98}	degradation of Mdm2
t_{14}	transcription from DNA to p53 mRNA transcript	t_{99}	degradation of Mdm2p
t_{15}	degradation of p53 mRNA	t_{100}	transition of IκBα from nucleus to cytoplasm
t_{16}	translation from p53 mRNA to p53n	t_{101}	transition of IκBα from cytoplasm to nucleus
t_{17}	transcription from DNA to Mdm2 mRNA transcript	t_{102}	creation of complex IκBαn and NF-κBn
t_{18}	transcription from DNA to PTEN mRNA transcript	t_{103}	transition of IκBα and NF-κB complex from nucleus to cytoplasm
t_{19}	degradation of Mdm2 mRNA	t_{104}	degradation of IκBα
t_{20}	degradation of PTEN mRNA	t_{105}	creation of complex IκBα and NF-κB
t_{21}	translation from PTEN mRNA to PTEN	t_{106}	IκBα and NF-κB complex degaradation
t_{22}	degradation of PTEN	t_{107}	transition of NF-κB from cytoplasm to nucleus
t_{23}	dephosphorylation of PIP3 by PTEN	t_{108}	source of IKK
t_{24}	phosphorylation of PIP2	t_{109}	degradation of IκBα after degradation of complex
t_{25}	dephosphorylation of AKTp	t_{110}	transcription to Wip1 mRNA transcript
t_{26}	phosphorylation of AKT by PIP3 and ATMan	t_{111}	degradation of Wip1 mRNA by shRNA

No.	Biological meaning	No.	Biological meaning
t_{27}	phosphorylation of Mdm2 by AKTp	t_{112}	synthesis of shRNA
t_{28}	dephosphorylation of Mdm2p	t_{113}	transition of KSRPpn from nucleus to cytoplasm
t_{29}	degradation of Mdm2p by Chk2p	t_{114}	spontaneous phosphorylation of p53n
t_{30}	degradation of Mdm2 by Chk2pn	t_{115}	uncoupling of IκBα and NF-κB complex
t_{31}	translation from Mdm2 mRNA to Mdm2	t_{116}	phosphorylation of IκBα by IKKa
t_{32}	translation from Wip1 mRNA to Wip1n	t_{117}	degradation of IκBαp
t_{33}	degradation of Wip1n	t_{118}	phosphorylation of IκBα in complex with NF-κB by IKKa
t_{34}	degradation of Wip1 mRNA	t_{119}	IκBαp and NF-κB complex degaradation
t_{35}	transcription from DNA to Chk2 mRNA transcript	t_{120}	degradation of IκBαp after degradation of complex
t_{36}	degradation of Chk2 mRNA	t_{121}	spontaneous inactivation of TNFR1
t_{37}	translation from Chk2 mRNA to Chk2n	t_{122}	independent activation of GATM
t_{38}	dephosphorylation of Chk2pn by Wip1n	t_{123}	p53pn dependent activation of GATM
t_{39}	phosphorylation of Chk2n by ATMan	t_{124}	CREBpn dependent activation of GATM
t_{40}	degradation of Chk2n	t_{125}	activation of GCHK2
t_{41}	degradation of Chk2pn	t_{126}	activation of GPTEN by p53pn
t_{42}	transcription to ATM mRNA transcript	t_{127}	p53pn dependent activation of GMdm2
t_{43}	translation from ATM mRNA to ATMn	t_{128}	p53pn dependent activation of Gp21
t_{44}	degradation of ATMn	t_{129}	p53pn dependent activation of GBax
t_{45}	degradation of ATM mRNA	t_{130}	NF-κBn dependent activation of GA20
t_{46}	degradation of Wip1 mRNA by miR 16	t_{131}	inactivation of GATM
t_{47}	maturation of pre miR 16	t_{132}	inactivation of GCHK2
t_{48}	transcription to pre mir 16 transcript by KSRPpn	t_{133}	inactivation of GPTEN
t_{49}	degradation of miR 16	t_{134}	inactivation of GMdm2
t_{50}	degradation of pre miR 16	t_{135}	inactivation of Gp21
t_{51}	dephosphorylation of ATMpn by Wip1n	t_{136}	inactivation of GBax
t_{52}	phosphorylation of ATMn by DSB	t_{137}	inactivation of GA20
t_{53}	degradation of ATMpn	t_{138}	inactivation of GIκBα
t_{54}	transition of ATMpn to ATMan by MRNpn	t_{139}	NF-κBn dependent activation of GIκBα
t_{55}	transition of ATMan to ATMpn by Wip1n	t_{140}	independent activation of GWip1
t_{56}	degradation of ATMan	t_{141}	inactivation of GWip1
t_{57}	dephosphorylation of CREBpn	t_{142}	p53pn dependent activation of GWip1
t_{58}	phosphorylation of CREBn by ATMan	t_{143}	NF-κBn dependent activation of GWip1
t_{59}	phosphorylation of MRNn by DSB	t_{144}	CREBpn dependent activation of GWip1
t_{60}	dephosphorylation of MRNpn	t_{145}	source of NF-κB
t_{61}	phosphorylation of MRNn by ATMpn	t_{146}	inactivation of Gp53
t_{62}	phosphorylation of KSRP by ATMan	t_{147}	NF-κBn dependent activation of Gp53
t_{63}	dephosphorylation of KSRPp	t_{148}	reactivation of GA20
t_{64}	transition of KSRPp from cytoplasm to nucleus	t_{149}	reactivation of GATM
t_{65}	transition of Mdm2p from cytoplasm to nucleus	t_{150}	reactivation of GBax
t_{66}	degradation of Mdm2pn by Chk2pn	t_{151}	reactivation of GCHK2
t_{67}	phosphorylation of Mdm2pn by ATMan	t_{152}	reactivation of GIκBα
t_{68}	dephosphorylation of Mdm2ppn by Wip1n	t_{153}	reactivation of GMdm2
t_{69}	degradation of Mdm2ppn by Chk2pn	t_{154}	reactivation of Gp21
t_{70}	phosphorylation of p53n by ATMan	t_{155}	reactivation of Gp53
t_{71}	inhibition of Chk2 mRNA by p53pn	t_{156}	reactivation of GPTEN
t_{72}	source of TNFα	t_{157}	reactivation of GWip1
t_{73}	source of TNFR1	t_{158}	creation of pool of PIP3
t_{74}	activation of TNFR1 by TNFα	t_{159}	creation of pool of AKTp
t_{75}	transition of IKKK to IKKKa by TNFR1	t_{160}	creation of pool of KSRPpn
t_{76}	transition of IKKKa to IKKK	t_{161}	creation of pool of CREBpn after activation of GATM
t_{77}	transcription from DNA to A20 mRNA transcript by IκBαn	t_{162}	creation of pool of MRNpn
t_{78}	translation from A20 mRNA to A20	t_{163}	creation of pool of IKKKa
t_{79}	degradation of A20 mRNA	t_{164}	creation of pool of CREBpn after activation of GWip1

No.	Biological meaning	No.	Biological meaning
t_{80}	transcription from DNA to IκBα mRNA transcript	t_{165}	spontaneous activation of GBax
t_{81}	degradation of A20	t_{166}	spontaneous activation of Gp21
t_{82}	degradation of IκBα mRNA	t_{167}	spontaneous activation of GMdm2
t_{83}	translation from IκBα mRNA to IκBα	t_{168}	spontaneous activation of GPTEN
t_{84}	transition of IKKa to IKKi by A20	t_{169}	spontaneous activation of Gp53

Supplementary Table S3. Significance analysis for the proposed Petri net model with two excitations (541 t-inv).

Significance analysis			
Model of ATM-p53-NF-κB with presence of TNF & IR (541 t-inv)			
no.	name of transition	t-inv	frequency trans/t-inv
t_1	creation of DSB stimulated by IR	418	77,26%
t_2	source of IR	418	77,26%
t_{42}	transcription to ATM mRNA transcript	418	77,26%
t_{13}	source of DNA	416	76,89%
t_{14}	transcription from DNA to p53 mRNA transcript	416	76,89%
t_{52}	phosphorylation of ATMn by DSB	415	76,71%
t_{16}	translation from p53 mRNA to p53n	414	76,52%
t_{43}	translation from ATM mRNA to ATMn	414	76,52%
t_{54}	transition of ATMpn to ATMan by MRNpn	407	75,23%
t_{162}	creation of pool of MRNpn	407	75,23%
t_{107}	transition of NF-κB from cytoplasm to nucleus	259	47,87%
t_{145}	source of NF-κB	258	47,69%
t_{39}	phosphorylation of Chk2n by ATMan	222	41,04%
t_{35}	transcription from DNA to Chk2 mRNA transcript	208	38,45%
t_{125}	activation of GCHK2	208	38,45%
t_{147}	NF-κBn dependent activation of Gp53	208	38,45%
t_{169}	spontaneous activation of Gp53	208	38,45%
t_{37}	translation from Chk2 mRNA to Chk2n	201	37,15%
t_{122}	independent activation of GATM	176	32,53%
t_{124}	CREBpn dependent activation of GATM	176	32,53%
t_{161}	creation of pool of CREBpn after activation of GATM	176	32,53%
t_{17}	transcription from DNA to Mdm2 mRNA transcript	166	30,68%
t_{31}	translation from Mdm2 mRNA to Mdm2	155	28,65%
t_{110}	transcription to Wip1 mRNA transcript	155	28,65%
t_{114}	spontaneous phosphorylation of p53n	145	26,80%
t_{10}	phosphorylation of p53n by Chk2pn	140	25,88%
t_{70}	phosphorylation of p53n by ATMan	140	25,88%
t_{127}	p53pn dependent activation of GMdm2	130	24,03%
t_{27}	phosphorylation of Mdm2 by AKTp	127	23,48%
t_{159}	creation of pool of AKTp	127	23,48%
t_{32}	translation from Wip1 mRNA to Wip1n	116	21,44%
t_{142}	p53pn dependent activation of GWip1	98	18,11%
t_{65}	transition of Mdm2p from cytoplasm to nucleus	97	17,93%
t_{123}	p53pn dependent activation of GATM	66	12,20%
t_{67}	phosphorylation of Mdm2pn by ATMan	62	11,46%
t_{167}	spontaneous activation of GMdm2	36	6,65%
t_{18}	transcription from DNA to PTEN mRNA transcript	33	6,10%
t_{126}	activation of GPTEN by p53pn	30	5,55%
t_{80}	transcription from DNA to IκBα mRNA transcript	29	5,36%
t_{139}	NF-κBn dependent activation of GIκBα	29	5,36%
t_{38}	dephosphorylation of Chk2pn by Wip1n	26	4,81%
t_{68}	dephosphorylation of Mdm2ppn by Wip1n	26	4,81%
t_9	dephosphorylation of p53pn by Wip1n	25	4,62%
t_4	transcription from DNA to p21 mRNA transcript	22	4,07%
t_{21}	translation from PTEN mRNA to PTEN	22	4,07%
t_3	transcription from DNA to Bax mRNA transcript	20	3,70%
t_{11}	degradation of p53pn by Mdm2pn	20	3,70%
t_{128}	p53pn dependent activation of Gp21	20	3,70%
t_{129}	p53pn dependent activation of GBax	20	3,70%
t_{140}	independent activation of GWip1	19	3,51%
t_{143}	NF-κBn dependent activation of GWip1	19	3,51%
t_{144}	CREBpn dependent activation of GWip1	19	3,51%
t_{164}	creation of pool of CREBpn after activation of GWip1	19	3,51%
t_{29}	degradation of Mdm2p by Chk2p	18	3,33%

Significance analysis			
Model of ATM-p53-NF-κB with presence of TNF & IR (541 t-inv)			
no.	name of transition	t-inv	frequency trans/t-inv
t_{30}	degradation of Mdm2 by Chk2pn	18	3,33%
t_{66}	degradation of Mdm2pn by Chk2pn	18	3,33%
t_{69}	degradation of Mdm2ppn by Chk2pn	18	3,33%
t_{77}	transcription from DNA to A20 mRNA transcript by IκBαn	18	3,33%
t_{83}	translation from IκBα mRNA to IκBα	18	3,33%
t_{96}	degradation of Mdm2ppn	18	3,33%
t_{130}	NF-κBn dependent activation of GA20	18	3,33%
t_{89}	transition of IKK to IKKa by ATMan	16	2,96%
t_{48}	transcription to pre mir 16 transcript by KSRPpn	15	2,77%
t_{108}	source of IKK	15	2,77%
t_{160}	creation of pool of KSRPpn	15	2,77%
t_{47}	maturation of pre miR 16	14	2,59%
t_{33}	degradation of Wip1n	13	2,40%
t_{34}	degradation of Wip1 mRNA	13	2,40%
t_{46}	degradation of Wip1 mRNA by miR 16	13	2,40%
t_{51}	dephosphorylation of ATMpn by Wip1n	13	2,40%
t_{55}	transition of ATMan to ATMpn by Wip1n	13	2,40%
t_{111}	degradation of Wip1 mRNA by shRNA	13	2,40%
t_{112}	synthesis of shRNA	13	2,40%
t_{12}	degradation of p53n by Mdm2pn	12	2,22%
t_{5}	translation from p21 mRNA to p21	11	2,03%
t_{6}	translation from Bax mRNA to Bax	11	2,03%
t_{7}	degradation of p21	11	2,03%
t_{8}	degradation of Bax	11	2,03%
t_{19}	degradation of Mdm2 mRNA	11	2,03%
t_{20}	degradation of PTEN mRNA	11	2,03%
t_{22}	degradation of PTEN	11	2,03%
t_{23}	dephosphorylation of PIP3 by PTEN	11	2,03%
t_{24}	phosphorylation of PIP2	11	2,03%
t_{92}	degradation of p21 mRNA	11	2,03%
t_{93}	degradation of Bax mRNA	11	2,03%
t_{97}	degradation of Mdm2pn	11	2,03%
t_{98}	degradation of Mdm2	11	2,03%
t_{99}	degradation of Mdm2p	11	2,03%
t_{0}	damage repair induced by p53pn	10	1,85%
t_{71}	inhibition of Chk2 mRNA by p53pn	10	1,85%
t_{90}	inhibition of transcription of IκBα mRNA by p53pn	10	1,85%
t_{91}	inhibition of transcription of A20 mRNA by p53pn	10	1,85%
t_{94}	degradation of p53pn	10	1,85%
t_{118}	phosphorylation of IκBα in complex with NF-κB by IKKa	10	1,85%
t_{119}	IκBαp and NF-κB complex degaradation	10	1,85%
t_{120}	degradation of IκBαp after degradation of complex	10	1,85%
t_{101}	transition of IκBα from cytoplasm to nucleus	8	1,48%
t_{78}	translation from A20 mRNA to A20	7	1,29%
t_{102}	creation of complex IκBαn and NF-κBn	7	1,29%
t_{103}	transition of IκBα and NF-κB complex from nucleus to cytoplasm	7	1,29%
t_{105}	creation of complex IκBα and NF-κB	7	1,29%
t_{60}	dephosphorylation of MRNpn	5	0,92%
t_{84}	transition of IKKa to IKKi by A20	5	0,92%
t_{85}	transition of IKKi to IKKii	5	0,92%
t_{86}	transition of IKKii to IKK	5	0,92%
t_{116}	phosphorylation of IκBα by IKKa	5	0,92%
t_{117}	degradation of IκBαp	5	0,92%
t_{25}	dephosphorylation of AKTp	4	0,74%
t_{26}	phosphorylation of AKT by PIP3 and ATMan	4	0,74%
t_{41}	degradation of Chk2pn	4	0,74%
t_{44}	degradation of ATMn	4	0,74%
t_{45}	degradation of ATM mRNA	4	0,74%
t_{53}	degradation of ATMpn	4	0,74%
t_{56}	degradation of ATMan	4	0,74%
t_{57}	dephosphorylation of CREBpn	4	0,74%
t_{58}	phosphorylation of CREBn by ATMan	4	0,74%
t_{61}	phosphorylation of MRNn by ATMpn	4	0,74%
t_{62}	phosphorylation of KSRP by ATMan	4	0,74%
t_{63}	dephosphorylation of KSRPp	4	0,74%
t_{87}	transition of IKK to IKKa by IKKKa	4	0,74%

Significance analysis			
Model of ATM-p53-NF-κB with presence of TNF & IR (541 t-inv)			
no.	name of transition	t-inv	frequency trans/t-inv
t_{158}	creation of pool of PIP3	4	0,74%
t_{163}	creation of pool of IKKKa	4	0,74%
t_{72}	source of TNFα	3	0,55%
t_{74}	activation of TNFR1 by TNFα	3	0,55%
t_{168}	spontaneous activation of GPTEN	3	0,55%
t_{15}	degradation of p53 mRNA	2	0,37%
t_{73}	source of TNFR1	2	0,37%
t_{95}	degradation of p53n	2	0,37%
t_{106}	IκBα and NF-κB complex degaradation	2	0,37%
t_{109}	degradation of IκBα after degradation of complex	2	0,37%
t_{115}	uncoupling of IκBα and NF-κB complex	2	0,37%
t_{165}	spontaneous activation of GBax	2	0,37%
t_{166}	spontaneous activation of Gp21	2	0,37%
t_{28}	dephosphorylation of Mdm2p	1	0,18%
t_{36}	degradation of Chk2 mRNA	1	0,18%
t_{40}	degradation of Chk2n	1	0,18%
t_{49}	degradation of miR 16	1	0,18%
t_{50}	degradation of pre miR 16	1	0,18%
t_{59}	phosphorylation of MRNn by DSB	1	0,18%
t_{64}	transition of KSRPp from cytoplasm to nucleus	1	0,18%
t_{75}	transition of IKKK to IKKKa by TNFR1	1	0,18%
t_{76}	transition of IKKKa to IKKK	1	0,18%
t_{79}	degradation of A20 mRNA	1	0,18%
t_{81}	degradation of A20	1	0,18%
t_{82}	degradation of IκBα mRNA	1	0,18%
t_{88}	inhibition of Ra by A20	1	0,18%
t_{100}	transition of IκBα from nucleus to cytoplasm	1	0,18%
t_{104}	degradation of IκBα	1	0,18%
t_{113}	transition of KSRPpn from nucleus to cytoplasm	1	0,18%
t_{121}	spontaneous inactivation of TNFR1	1	0,18%
t_{131}	inactivation of GATM	1	0,18%
t_{132}	inactivation of GCHK2	1	0,18%
t_{133}	inactivation of GPTEN	1	0,18%
t_{134}	inactivation of GMdm2	1	0,18%
t_{135}	inactivation of Gp21	1	0,18%
t_{136}	inactivation of GBax	1	0,18%
t_{137}	inactivation of GA20	1	0,18%
t_{138}	inactivation of GIκBα	1	0,18%
t_{141}	inactivation of GWip1	1	0,18%
t_{146}	inactivation of Gp53	1	0,18%
t_{148}	reactivation of GA20	1	0,18%
t_{149}	reactivation of GATM	1	0,18%
t_{150}	reactivation of GBax	1	0,18%
t_{151}	reactivation of GCHK2	1	0,18%
t_{152}	reactivation of GIκBα	1	0,18%
t_{153}	reactivation of GMdm2	1	0,18%
t_{154}	reactivation of Gp21	1	0,18%
t_{155}	reactivation of Gp53	1	0,18%
t_{156}	reactivation of GPTEN	1	0,18%
t_{157}	reactivation of GWip1	1	0,18%

Supplementary Table S4. List of parameters of the ATM/p53/NF-κB signaling pathway model described in [Jonak *et al.*, 2016].

No.	Biological meaning	No.	Biological meaning
1	Total number of AKT	63	IkBa in complex with NFkB degradation
2	Total number of CREB	64	IkBa-NFkB association rate
3	Total number of KMRN	65	MM for Wip1 influence on NFkB dep. genes
4	Total number of KSRP	66	MM for IKKK inhibition by A20
5	Total number of PIP	67	MM for IKKa inactivation by A20
6	p53 spontaneous activation	68	MM for IKKa inactivation by A20
7	p53 activation by ATM	69	NFkB dependent genes activation

No.	Biological meaning	No.	Biological meaning
8	p53 activation by Chk2	70	IkBa and A20 genes inactivation
9	Mdm2 phosphorylation by AKT	71	A20 and IkBa mRNA synthesis
10	Nuclear Mdm2 reactivation by Wip1	72	IkBa translation
11	Nuclear Mdm2 inactivation by ATM	73	DSB damage caused by IR
12	PIP3 activation	74	Apoptotic DSB degradation
13	AKT activation by PIP	75	ATM activation by DSB formation
14	AKT activation by ATM	76	ATM activation by MRN complex formation
15	p53 inactivation by Wip1	77	Chk2 and CREB activation by ATM
16	Mdm2 dephosphorylation rate	78	MRN complex activation by ATM
17	PIP inactivation by PTEN	79	MRN complex activation by DSB
18	AKT inactivation	80	DSB repair rate
19	p53 transcript degradation	81	ATM inactivation by Wip1
20	p53 protein spontaneous degradation	82	Chk2 inactivation by Wip1
21	p53 degradation by Mdm2	83	MRN complex inactivation
22	p53p protein spontaneous degradation	84	CREB complex inactivation
23	p53p degradation by Mdm2	85	ATM transcript degradation
24	Mdm2 transcript degradation	86	ATM protein degradation
25	Mdm2 protein spontaneous degradation	87	Chk2 transcript degradation
26	Mdm2p protein spontaneous degradation	88	Chk2 protein degradation
27	Mdm2 degradation by Chk2	89	MM for DSB repair
28	PTEN transcript degradation rate	90	MM for ATM activation by DSB
29	PTEN degradation rate	91	MM for MRN complex activation by DSB
30	Mdm2 nuclear import	92	Chk2 and ATM gene activation
31	MM for p53 activation by ATM	93	Wip1 and ATM gene activation by CREB
32	MM for Chk2 influence on the system	94	Chk2 gene inactivation
33	MM for p53 caused translation inhibition	95	ATM synthesis
34	p53 gene spontaneous activation	96	Chk2 synthesis
35	Spont. activation of Mdm2 and PTEN genes	97	ATM transcription
36	p53-dep. activation of Mdm2, PTEN, ATM and Wip1 genes	98	Chk2 transcription
37	Mdm2 and PTEN genes inactivation	99	KSRP activation
38	Synthesis of p53 mRNA	100	miR-16 maturation
39	Synthesis of Mdm2 mRNA	101	KSRP deactivation
40	Synthesis of PTEN mRNA	102	Wip1 mRNA spontaneous degradation
41	p53 translation rate	103	Wip1 mRNA degradation caused by siRNA
42	Mdm2 translation rate	104	Wip1 mRNA degradation caused by miR-16
43	PTEN translation rate	105	Wip1 protein degradation rate
44	Total number of receptors	106	Wip1 mRNA degradation caused by miR-16
45	IKKa med. IkBa in complexes phosphphorylation	107	Wip1 mRNA degradation caused by miR-16
46	IKKa mediated IkBa phosphorylation	108	KSRP nuclear export
47	IKKK activation	109	KSRP nuclear import
48	IKKii transformation	110	Wip1 gene deactivation
49	IKK activation	111	WIP1 mRNA synthesis rate
50	Activation of IKK by ATM	112	pre-miR-16 production rate
51	TNF caused receptor activation	113	WIP1 translation rate
52	IKKK inactivation rate	114	Bax mRNA degradation
53	IKK inactivation rate	115	Bax degradation
54	Receptor inactivation rate	116	Bax mRNA synthesis
55	Phosphorylated IkBa degradation	117	Bax translation
56	A20 and IkBa transcript degradation	118	p21t degradation rate
57	Free IkBa degradation	119	p21 degradation rate
58	A20 degradation rate	120	p21 mRNA synthesis
59	IkBa nuclear export	121	p21 translation
60	IkBa-NFkB nuclear export	122	A20 translation
61	NFkB nuclear import	123	p53 gene spontaneous inactivation
62	IkBa nuclear import		

ODE model simulations and analysis. The simulations and analysis of the ODE model were performed in Matlab R2020b. The following files were attached to the publication:

- ATMmodelD.m - model described by ordinary differential equations (ODE).
- ATMparametersD.m - ODE model parameters.
- ATMinitial_cD.m - the initial conditions of simulation.
- ATMsimulationD.m - main file that enables simulation and sensitivity analysis of the ODE model.

References

[Jonak *et al.*, 2016] Jonak, K., Kurpas, M., Szoltysek, K., Janus, P., Abramowicz, A., Puszynski, K. (2016) A novel mathematical model of ATM/p53/NF-κB pathways points to the importance of the DDR switch-off mechanisms. *BMC Systems Biology*, **10(1)**, 75.
